# Multiplex detection and application of MALDI-TOF NAMS for porcine diarrheal pathogens

**DOI:** 10.1128/spectrum.01328-25

**Published:** 2025-10-27

**Authors:** Jiangbing Shuai, Shiqi Song, Xiao Han, Ya Zhao, Kexin Chen, Youran Guo, Huimin Guo, Nan Zhang, Xiaofeng Zhang

**Affiliations:** 1 Hangzhou Customs Technical Center, Hangzhou, China; 2 Zhejiang Academy of Science & Technology for Inspection & Quarantine 91629 , Hangzhou, China; 3 Zhejiang Digena Diagnostic Technology Co., Ltd., Hangzhou, China; 4 Department of Veterinary Medicine, College of Animal Sciences, Zhejiang University 12377, Hangzhou, China; 5 College of Life Sciences, China Jiliang University 92270, Hangzhou, China; Barnard College, Columbia University, New York, USA

**Keywords:** porcine diarrheal pathogens, MALDI-TOF NAMS, multiplex detection

## Abstract

Porcine diarrheal diseases caused by mixed viral and bacterial infections pose significant challenges to swine health and production. Rapid and accurate identification of key pathogens, including porcine epidemic diarrhea virus (PEDV), transmissible gastroenteritis virus (TGEV), porcine deltacoronavirus (PDCoV), porcine rotavirus (PoRV), swine acute diarrhea syndrome coronavirus (SADS-CoV), porcine bocavirus (PBoV), hepatitis E virus (HEV), and *Salmonella*, remains a challenge due to overlapping clinical symptoms and co-infections. The zoonotic potential of HEV and PBoV also highlights the need for reliable, high-throughput detection methods. We developed a multiplex Matrix-Assisted Laser Desorption/Ionization Time-of-Flight Nucleic Acid Mass Spectrometry (MALDI-TOF NAMS) assay for the simultaneous detection of eight major porcine gastrointestinal pathogens. The method utilizes a multiplex PCR approach and a single-base extension strategy to improve sensitivity and specificity, and the extension products are displayed by mass spectrometry. The analytical performance of the assay was assessed by evaluating the limits of detection (LoD), analytical specificity, and repeatability. Additionally, we validated its diagnostic accuracy using 242 clinical samples, comparing the results with real-time quantitative PCR (qPCR) as the reference method. The MALDI-TOF NAMS assay demonstrated high analytical specificity, with no cross-reactivity to non-target pathogens. The limit of detection (LoD) ranged from 12.20 to 33.59 copies/μL. The intra- and inter-batch reproducibility assessments showed a 100% detection rate across high, medium, and low concentrations (20/20 per concentration, 60/60 total). Validation using 242 clinical samples demonstrated a 98.3% sensitivity and 99.5% specificity compared to qPCR, with an overall concordance rate of 96.2%. The MALDI-TOF NAMS assay provides a sensitive and high-throughput method for the detection and genotyping of major swine diarrhea pathogens. This method performs well in mixed infections and allows for expansion of pathogen species, making it an important addition to traditional diagnostic methods.

**IMPORTANCE:**

Porcine diarrheal diseases are a major threat to the swine industry, often caused by multiple viruses and bacteria infecting animals at the same time. Fast and accurate detection of these pathogens is crucial to prevent outbreaks, reduce economic losses, and protect public health, especially given the potential of some pathogens to infect humans. This study introduces a new method that uses nucleic acid mass spectrometry technology to quickly and accurately detect eight major pathogens from pig samples, such as feces, blood, or tissue. Unlike traditional tests that often detect one pathogen at a time, this method can screen for many at once, even in complex cases of mixed infections. It is sensitive, reliable, and can handle large numbers of samples efficiently. This tool offers farmers, veterinarians, and disease control agencies a faster and more effective way to monitor pig health and respond to outbreaks before they spread.

## INTRODUCTION

Porcine gastrointestinal diseases are among the most common health issues in swine production and carry significant economic consequences. These diarrheal diseases are particularly common in piglets, where viral and bacterial diarrhea can lead directly to growth retardation and even high mortality due to the incomplete development of their digestive system (1). Notable among these are porcine epidemic diarrhea virus (PEDV), transmissible gastroenteritis virus (TGEV), porcine delta coronavirus (PDCoV), porcine rotavirus (PoRV), and swine acute diarrhea syndrome coronavirus (SADS-CoV). These gastrointestinal viruses are highly detrimental, and their predominantly clinical symptoms, diarrhea, are often indistinguishable, posing significant challenges to differential diagnosis in the field (2, 3). Porcine bocavirus (PBoV), a virus frequently detected in pigs, has recently emerged as a potential contributor to gastrointestinal diseases (4). Increasing evidence suggests that PBoV is often present in mixed infections with other pathogens, such as PEDV, PCV2, and CSFV, where it can exacerbate clinical severity and increase herd morbidity and mortality (5). Although genotypes G1, G2, and G3 have been identified, studies investigating their prevalence and significance in diarrheic pigs remain limited (6). Beyond swine health, PBoV has also been sporadically detected in humans, raising concern about its zoonotic potential (7). These findings underscore the importance of including PBoV in multiplex diagnostic assays to improve the etiological diagnosis of porcine diarrheal diseases. Additionally, fecal-oral pathogens, such as *Salmonella* and hepatitis E virus (HEV), infect pigs and pose serious public health concerns due to their zoonotic potential (8, 9). HEV and *Salmonella* excreted in the feces of infected pigs can contaminate the environment, leading to cross-species transmission to humans and amplifying the risk of foodborne or waterborne outbreaks.

Fast and accurate detection of these pathogens is crucial to prevent outbreaks, reduce economic losses, and protect public health, especially given the potential of some pathogens to infect humans. Importantly, epidemiological studies have shown that porcine coronaviruses (PEDV, PDCoV, TGEV, and SADS-CoV) and rotavirus (PoRV) frequently co-circulate, with mixed or secondary infections significantly increasing herd morbidity and mortality (10). Because these pathogens are serologically distinct, diagnosing them individually increases both cost and turnaround time. Multiplex detection can improve diagnostic efficiency, identify the major causative agents in co-infections, and facilitate the timely removal or management of infected animals, thereby reducing herd-level transmission and economic loss (11). From a broader One Health perspective, comprehensive pathogen detection is also critical for surveillance, outbreak control, and informing vaccination strategies, while also reducing the risk of zoonotic spread (12).

Currently, diagnostic methods for porcine gastrointestinal pathogens include ELISA, real-time PCR (qPCR), and next-generation sequencing (NGS). ELISA is suitable for serological surveillance but may fail in early-stage infections and is less reliable in the presence of antigenic cross-reactivity. While qPCR is widely used and offers high sensitivity and specificity, it is often limited in multiplexing capacity and requires separate reactions to detect multiple pathogens, increasing cost and time. NGS provides a broad pathogen overview but is costly, labor-intensive, and less practical for routine diagnostics. In contrast, Matrix-Assisted Laser Desorption/Ionization Time-of-Flight Nucleic Acid Mass Spectrometry (MALDI-TOF NAMS) offers the advantage of simultaneously detecting multiple pathogens in a single reaction, with high sensitivity, excellent specificity, and robust performance in complex samples, including those with mixed infections.

MALDI-TOF NAMS is a nucleic acid detection technique that combines multiplex PCR with mass spectrometry (13, 14). It operates by ionizing nucleic acid fragments and measuring their unique time-of-flight in a vacuum to differentiate target DNA sequences based on their molecular weights. By identifying distinct unextended probes (UEPs) and single-base extended products (SEPs), this technology can simultaneously detect and analyze several or even dozens of target sequences in the same sample. This effectively improves the ability to detect multiple pathogens in a single reaction. MALDI-TOF NAMS, based on the sensitivity of PCR and utilizing the accuracy of mass spectrometry technology, can intuitively and rapidly present the results of multi-target detection. Originally, MALDI-TOF NAMS was employed in clinical diagnostics to detect disease- or drug resistance-related genes and served as a validation platform when discrepancies arose between first- and second-generation sequencing results (15, 16). The scalability and speed of MALDI-TOF NAMS make it particularly suitable for high-throughput screening in clinical and surveillance settings. It has been successfully applied to multiplex detection of various infectious agents, such as human coronaviruses (17), sexually transmitted pathogens (18), *Mycobacterium tuberculosis* (19), and swine viruses (20).

This study aims to address this gap by developing a MALDI-TOF NAMS assay for the simultaneous detection of eight major pathogens associated with porcine diarrheal syndromes: PDCoV, PEDV, TGEV, SADS-CoV, HEV, PoRV, PBoV, and Sal. By enabling the rapid, accurate, and high-throughput detection of these pathogens, this assay not only supports the timely prevention and control of porcine infectious diarrheal diseases but also provides an effective tool for pathogen surveillance in swine populations. Furthermore, from the perspective of cross-border animal trade and biosecurity, such a method holds promise for enhancing the diagnostic capabilities of customs and quarantine agencies, ensuring the early detection and monitoring of pathogens that may pose risks to animal health, food safety, and public health.

## MATERIALS AND METHODS

### Viruses and positive templates

The strains, as well as bacterial and viral nucleic acid samples, used in this study were primarily employed for specificity evaluation. Inactivated viral strains, including Porcine epidemic diarrhea virus (PEDV, CV777 strain), Porcine transmissible gastroenteritis virus (TGEV, WH-1R strain), Porcine reproductive and respiratory syndrome virus (PRRSV, HuN4 strain), Pseudorabies virus (PRV, Bartha strain), and Porcine circovirus type 2 (PCV-2, JH-SRJ strain), were kindly provided by Zhejiang University. Positive nucleic acid samples of porcine deltacoronavirus (PDCoV, P25 GH7DQ0301 strain), swine acute diarrhea syndrome coronavirus (SADS-CoV), porcine rotavirus (PRoV), porcine bocavirus (PBoV), porcine hepatitis E virus (HEV), classical swine fever virus (CSFV, C strain), foot-and-mouth disease virus (FMDV, OHM/02 and AKT-111 strains), Japanese encephalitis virus (JEV, strain SA14–14-2), and the PEDV+TGEV bivalent live vaccine (WH-1R + AJ1102R), as well as nucleic acid samples of *Salmonella enterica subsp. enterica* (ATCC 9842, ATCC 13076, and ATCC 35640), were collected and preserved by our laboratory.

### Reagents and kits

Viral nucleic acids were extracted and purified using the TaKaRa MiniBEST Viral RNA/DNA Extraction Kit (Takara, Japan; Cat#9766). Multiplex PCR was performed using the Hifair V Multiplex One Step RT-PCR Kit (Yeasen, China; Cat#13089). dNTP digestion and extension reactions were performed using the IPLEX Universal Kit (Agena, USA). qPCR was performed using TB Green Premix Ex Taq (2×) (Takara, Japan; Cat#RR420). Clinical samples were subjected to nucleic acid extraction and purification using the MagNA Pure 24 Instrument and the corresponding Total NA Isolation Kit (Roche, Switzerland; Cat#07658036001).

### Primers and UEPs

Based on the genome sequences of each of the above pathogens retrieved from the NCBI database, primers for the conserved regions of the genes and corresponding unextended probes (UEPs) were designed. Sequence analysis and multiple sequence alignment were conducted using CLC Genomics Workbench 23 (Qiagen, Germany) and MEGA-X (Auckland, New Zealand). Primer and UEP designs were performed using Primer3 Plus (https://www.primer3plus.com) and MassARRAY Assay Design Suite (Agena, USA). For TGEV, primers were specifically designed to target the S gene, ensuring specificity by avoiding its PRCV variant. For HEV, a single-base extend site was designed to differentiate between HEV-3 (A, 5560.6 Da) and HEV-4 (C, 5536.6 Da) based on single nucleotide polymorphisms (SNPs) at the 3′ end (Fig. S1). PBoV genotypes (G1–G3) were determined from complete genome phylogenetic analysis (neighbor-joining, 1,000 bootstraps, Fig. S2). Genotype-specific primers and UEPs were then designed from conserved regions in either NS1 or VP1 to maximize inclusivity and specificity. Additionally, the porcine RPL4 gene (Accession number: XM_005659862) was used as the internal controls in this study.

To prevent potential peak interference caused by multiple primers in mass spectra, a 10-base tag (ACGTTGGGATG, highlighted in bold in Table 1) was appended to the 5′ end of each PCR primer. The primers and probes sequences were designed by the Primer-BLAST (https://www.ncbi.nlm.nih.gov/tools/primer-blast) and MFEprimer 4 (https://m4.igenetech.com). The sequences of all primers and UEPs are listed in Table 1. All oligonucleotides were synthesized and purified by Sangon Biotech (China).

**TABLE 1:** Primers and UEPs for MALDI-TOF NAMS^*a*^^,^^*b*^^,^^*c*^

Virus	Gene	PCR primer (5′→3′)^*d*^	UEP sequence	UEP (Da)	SEP (Da)
PBoV-G3	*NS1*	f: **acgttggatg**aaaagccacgctcatgcag	tgtttcccatcggta	4535.0	4806.2(A)
		r: **acgttggatg**ggtaacgccaaacgtgtttc			
PDCoV	*M*	f: **acgttggatg**catatcctgtggcggatttc	tacatgggcaagagc	4627.0	4954.1(T)
		r: **acgttggatg**cagtcgttaagcatggcaag			
Sal	*invA*	f: **acgttggatg**tagaacgaccccataaacac	acctatctggttgatt	4863.2	5190.3(T)
		r: **acgttggatg**tccattacctacctatctgg			
HEV	*ORF3*	f: **acgttggatg**tggttggatgaatataggg	gtggtttctggggtgac	5289.4	5536.6(C)5560.6(A)
		r: **acgttggatg**agtgccggcggtggtttctg			
PEDV	*N*	f: **acgttggatg**aaataaccagggtcgtggag	cattattattgcctcctc	5376.6	5703.8(A)
		r: **acgttggatg**tcttggactggttacgagac			
PBoV-G1	*NS1*	f: **acgttggatg**cccaacagttttcctctagc	ccacaaggtccttgagcg	5485.6	5756.8(T)
		r: **acgttggatg**agtagtgtgaggcaggtaac			
TGEV	*S*	f: **acgttggatg**tgtgatggagtatgggtatc	gggaacggttaaacgtagt	5917.8	6165(C)
		r: **acgttggatg**cattgtattgggattatgc			
PoRV	*VP6*	f: **acgttggatg**gtcaatcagactctacaag	cccagttactctacgtagcg	6076.0	6347.2(T)
		r: **acgttggatg**ggtcacatcctctcacta			
SADS-CoV	*N*	f: **acgttggatg**ggcttactctaaacccagtc	acactggggcatcagcattt	6430.2	6701.4(T)
		r: **acgttggatg**ttgggaaactggagtagctg			
PBoV-G2	*VP1*	f: **acgttggatg**gtgtttggttgtttgtccc	gggacccaatgcaagcatgga	6490.2	6761.4(A)
		r: **acgttggatg**gacacagtatggcaataccc			
Internal control	*RPL4*	f: **acgttggatg**tttggatctctgggcttttc	agatgctcaatacagaccttagc	7016.6	7287.8(A)
		r: **acgttggatg**ctgctaccctcaagagtaac			

^*a*^ Sorted by the molecular weight of the single-base extended product.

^*b*^ UEP extended single bases are marked with capital and underscores.

^*c*^ UEP, un-extended probe; SEP, single-base extended products; PDCoV, porcine deltacoronavirus; PEDV, porcine epidemic diarrhea virus; TGEV, transmissible gastroenteritis virus; SADS-CoV, swine acute diarrhea syndrome coronavirus; PoRV, porcine rotavirus; HEV-3/4, hepatitis E virus genotype 3/4; PBoV-G1/G2/G3, porcine bocavirus group 1/2/3.

^*d*^ The bolded regions within each primer sequence are fixed universal 10-base tags. These sequences ensure consistent extension initiation, optimize amplicon molecular weights for mass spectrometry analysis, and facilitate subsequent data processing.

### Construction of standard plasmids

The target gene sequences corresponding to the primer design regions for each virus were synthesized and cloned into the pUC-57 plasmid vector (Sangon Biotech, China). These recombinant plasmids served as standard templates to evaluate the performance of the detection system in this study. Details of the standard plasmids are listed in Table 2.

**TABLE 2:** Plasmids used in this study^*a*^

Plasmid name	Reference sequence	Amplification regions (bp)	Fragment length (bp)
pUC57_HEV-3	AF082843	5129 ~ 5508	380
pUC57_HEV-4	EF077630	5143–5522	380
pUC57_PBoV-G1	NC_016031	1979 ~ 2313	335
pUC57_PBoV-G2	NC_038537	4142 ~ 4555	414
pUC57_PBoV-G3	NC_024453	1784 ~ 2203	420
pUC57_PDCoV	MZ802774	275 ~ 654	380
pUC57_PEDV	FJ473389.1	262 ~ 701	440
pUC57_PoRV	EU372754	997 ~ 1356	360
pUC57_TGEV	HQ462571	20365 ~ 20789	425
pUC57_SADS-CoV	MT199592	26210 ~ 26609	400
pUC57_*Salmonella*	KR185982	1591 ~ 2040	450
pUC57_IC	XM_005659862	940 ~ 1437	498

^*a*^ PDCoV, porcine deltacoronavirus; PEDV, porcine epidemic diarrhea virus; TGEV, transmissible gastroenteritis virus; SADS-CoV, swine acute diarrhea syndrome coronavirus; PoRV, porcine rotavirus; HEV-3/4, hepatitis E virus genotype 3/4; PBoV-G1/G2/G3, porcine bocavirus group 1/2/3; IC, internal control.

The copy numbers of each plasmid were quantified using Qubit 4 (Thermo Fisher, USA). Each standard plasmid was initially diluted to 10^8^ copies/μL and then serially diluted tenfold to create a range of concentrations from 10^8^ to 10^0^ Copies/μL. For subsequent experiments, mixed plasmid solutions were prepared at each gradient concentration (10^7^ to 10^0^ Copies/μL) to serve as positive controls. Aliquots of these plasmid mixtures were stored for use in downstream assays.

### Optimization of reaction system and conditions

To optimize the reaction system, 10^4^ copies/μL plasmid templates of each target were tested with the primers and UEPs listed in Table 1. Subsequently, a mixed plasmid sample containing equal concentrations of 11 targets at 10^4^ copies/μL was used to optimize multiplex conditions. Initial concentrations of all primers and UEPs were set to 5 μmol/L, and adjustments were made to achieve uniform UEP peak intensities and an E_SEP/UEP_ ≥ 0.8. Optimization included fine-tuning primer and UEP concentrations, annealing temperatures (55°C–60°C), and annealing times (20–35 s) in the multiplex PCR system. Negative controls using ddH_2_O were included in all experiments, and the presence of correct mass spectrum peaks was used to evaluate the results.

### PCR setup and reaction conditions

Each reaction mixture contained 2 μL of recombinant plasmid (10^4^ copies/μL), 2.5 µL of 2 × Multiplex PCR buffer, 0.3 µL of enzyme mix, and 0.2 µL of each target-specific primer. The thermal cycling conditions were as follows: reverse transcription was performed at 50° for 10 min, initial denaturation at 95°C for 5 min, then 45 cycles of 95°C for 15 s, 60°C for 30 s, and finally 72°C for 5 min. After amplification, 2 µL of shrimp alkaline phosphatase (SAP) and reaction buffer were added to the PCR products for dNTP dephosphorylation. The mixture was incubated at 37°C for 40 min, followed by enzyme inactivation at 85°C for 5 min. For the extension reaction, the dephosphorylated products were mixed with 1 µL of UEP mix, 0.04 µL of Iplex enzyme, 0.2 µL of termination mix (containing ddNTP), 0.2 µL of Iplex buffer, and 0.56 µL of ddH_2_O. The extension program consisted of 40 cycles of 30 s at 95°C, 5 s at 95°C, 5 s at 52°C, 5 s at 80°C, and a final extension of 3 s at 72°C. The UEP will specifically bind to the target sequence in the PCR product, and the chain termination reaction using ddNTPs produced the SEP.

The SEPs were transferred to a 384-well plate, diluted to 25 µL with ddH_2_O, and centrifuged at 8,000 rpm for 2 min. The prepared plate, along with the inert matrix chip, was loaded into the DP-TOF mass spectrometer (Digena, China). Pre-edited assay files containing molecular weight information for each UEP and SEP were imported into the instrument software. After completing sample and plate setup, the resin was programmed to purify the products for mass spectrometry analysis. The results were evaluated based on the correct positioning of peaks in the mass spectrum. Detection of the corresponding UEP and SEP peaks for each target was used to confirm successful amplification and extension.

### Analytical specificity and sensitivity

Analytical specificity of NAMS was assessed by testing the assay against a panel of non-target pathogens to confirm absence of cross-reactivity. Non-target nucleic acids from inactivated CSFV, FMDV, PRRSV, PRV, and PCV-2 were used as negative controls, while a plasmid mixture at 10^4^ copies/μL served as the positive control, and ddH_2_O was used as the blank control.

The analytical sensitivity of NAMS was assessed by preparing a twofold serial dilution of the plasmid mixture (initial concentration: 100 copies/μL) to create a series of concentrations, including 50, 25, 12.5, 6.2, 3.1, and 1.6 copies/μL. The limit of detection (LoD) was defined as the lowest concentration at which all replicates (*n* = 10) tested positive.

### Repeatability and reproducibility

For repeatability evaluation, plasmid mixtures at high, medium, and low concentrations (10^6^, 10^4^, and 10^2^ copies/μL, respectively) were tested in 20 replicates under the same conditions. For reproducibility assessment, two additional independent experiments were conducted seven days apart, resulting in three independent experimental batches. Results from these batches were compared to evaluate consistency across different runs and time points.

### Evaluation of clinical sample results by MALDI-TOF NAMS

To validate the ability of the MALDI-TOF NAMS method to detect multiple pathogens in clinical samples, a total of 242 diarrhea samples of various types were evaluated using both the MALDI-TOF NAMS and qPCR methods. The samples included 97 fecal samples, 132 tissue samples, and 13 serum samples, all of which were collected from the Zhejiang Institute of Inspection and Quarantine Science and Technology between 2023 and 2024. All discordant results between the two methods were further analyzed using digital PCR (dPCR) for confirmation.

For sample preparation, blood samples (1–2 mL) were directly used for nucleic acid extraction. Fecal samples (1–2 g) were tenfold diluted with PBS, vortexed thoroughly, and centrifuged at 10,000 rpm for 5 min, after which the supernatant was collected. Tissue samples (1–2 g) were homogenized in 5–10 mL of 10 mM phosphate-buffered solution (PBS, pH 7.2) at a mass-to-volume ratio of 1:5. The homogenate was processed using a tissue grinder, followed by centrifugation at 10,000 rpm for 5 min, and the supernatant was collected.

Nucleic acid extraction and purification were performed using the MagNA Pure 24 Instrument and the corresponding kit. Subsequently, the optimized conditions for multiplex reverse transcription PCR, SAP digestion, and UEP extension were applied. Each sample was tested in triplicate, with ddH_2_O and a 10^4^ copies/μL plasmid mixture serving as negative and positive controls, respectively.

### qPCR reference assay

For comparison, commercial qPCR kits (Vipotion, China; Cat#SD-R-0411, SD-R-0511, SD-R-0611, SD-R-0911, MQ-R-5411) were used for the detection of PEDV, TGEV, PoRV, PDCoV, and HEV, according to the manufacturer’s instructions. For *Salmonella* and PBoV, published primers (Table S1) were used. qPCR was performed using TB Green Premix Ex Taq for single-cycle amplification, with a reaction volume of 20 µL containing 2 µL template, 10 µL master mix, and 0.2 µM primers. Cycling conditions were as follows: 95°C for 2 min; 40 cycles of 95°C for 10 s and 60°C for 30 s. Samples with Ct values below 38 were considered positive.

## RESULTS

### Single-target MALDI-TOF NAMS assay

**Fig 1:**
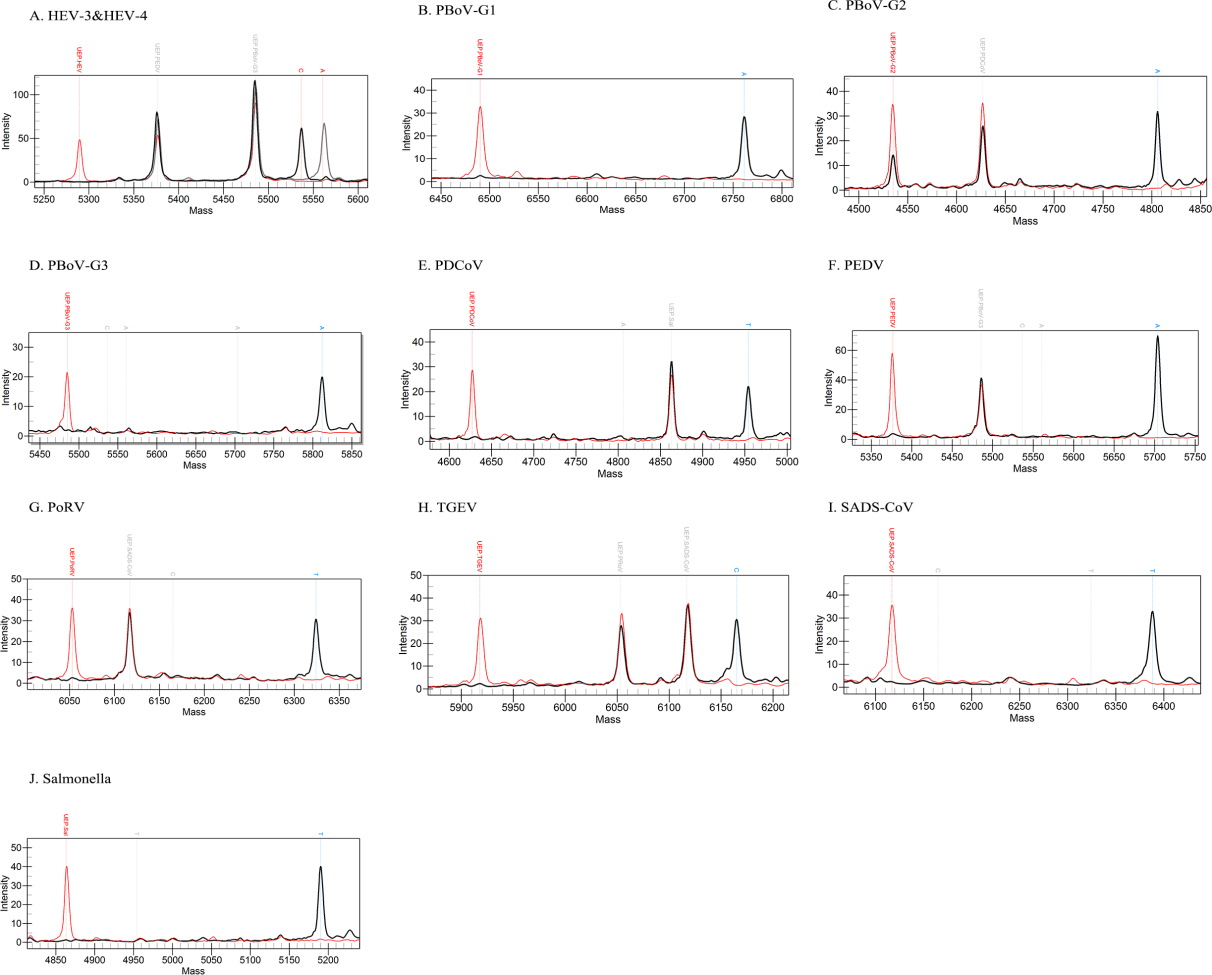
Superimposed MALDI-TOF NAMS spectra of 10^3^ copies/μL plasmid (black) and blank control (red) added for various pathogens. Panel **A to J** respectively show the mass spectrometry results of HEV-3 and HEV-4, PBoV-G1, PBoV-G2, PBoV-G3, PDCoV, PEDV, PoRV, TGEV, SADS-CoV, and *Salmonella*. The blank control for each target exhibits only UEP peaks, with no SEP peaks detected. In contrast, the plasmid shows extended SEP peaks, allowing clear differentiation of the presence or absence of the target nucleic acid fragment in the sample. UEP, un-extended probe; SEP, single-base extended products; PDCoV, porcine deltacoronavirus; PEDV, porcine epidemic diarrhea virus; TGEV, transmissible gastroenteritis virus; SADS-CoV, swine acute diarrhea syndrome coronavirus; PoRV, porcine rotavirus; HEV-3/4, hepatitis E virus genotype 3/4; PBoV-G1/G2/G3, porcine bocavirus group 1/2/3; Sal, *Salmonella*.

The mass spectrometry profiles using single plasmids as templates (Fig. 1) demonstrated that each tested standard plasmid produced a distinct SEP peak at the predicted mass position (black), while the corresponding blank controls detected only UEP peaks (red). The UEP and SEP peaks can be clearly distinguished for the specific mass of each target. The positions of both peaks were consistent with those listed in Table 1, confirming the specificity and functionality of the designed primers and UEPs.

### Multiplex detection

**Fig 2:**
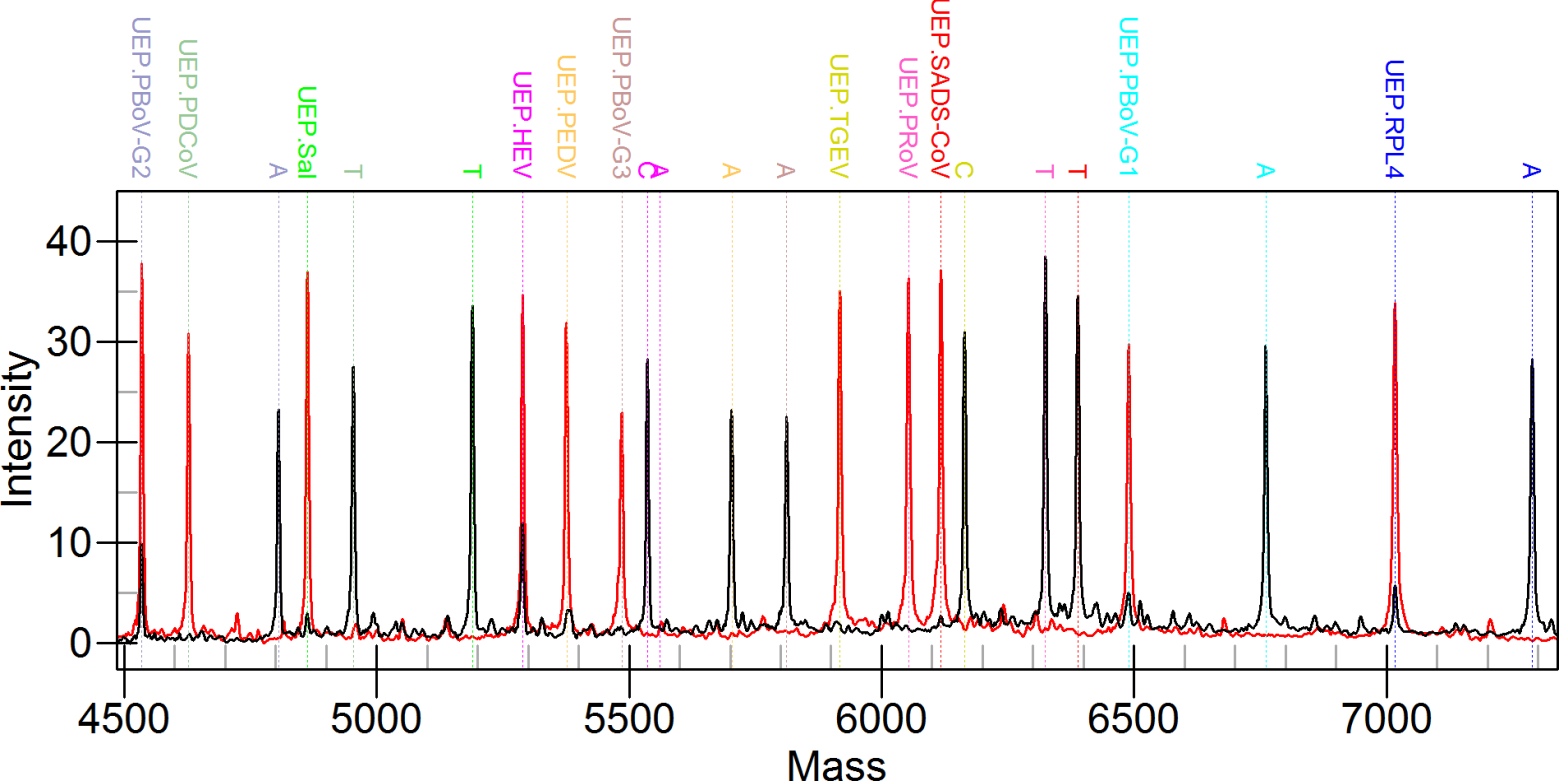
The black signal peak used 10^3^ copies/μL of the mixed plasmid as a template to simulate the presence of all targets. The red signal peaks were templated with ddH_2_O. The UEP and SEP of each target were labeled using their respective colors, where SEP was indicated using the bases (A, C, or T) extended by the target. The SEP peaks and UEP peaks can be clearly distinguished in the NAMS images. UEP, un-extended probe; SEP, single-base extended products; PDCoV, porcine deltacoronavirus; PEDV, porcine epidemic diarrhea virus; TGEV, transmissible gastroenteritis virus; SADS-CoV, swine acute diarrhea syndrome coronavirus; PoRV, porcine rotavirus; HEV-3/4, hepatitis E virus genotype 3/4; PBoV-G1/G2/G3, porcine bocavirus group 1/2/3; Sal, *Salmonella*.

Using a mixed plasmid template (10^4^ copies/μL for each target), the system’s ability to detect multiple targets simultaneously was evaluated. Figure 2 illustrates the mass spectra obtained under optimized reaction conditions. All 11 SEP peaks were detected at their expected mass positions, with minimal overlap or interference, demonstrating the successful multiplex amplification and extension of the UEPs. Additionally, no extraneous peaks or significant variations in peak height were observed, affirming the assay’s compatibility in a multiplex setting. To ensure uniform signal detection, the initial concentrations of primers and UEPs were adjusted during optimization (Table 3).

**TABLE 3:** Final concentrations of primers and UEPs used in MALDI-TOF NAMS^*a*^

Target	Primer (µmol/L)	UEP (µmol/L)
HEV	5	10
PBoV-G1	2	6
PBoV-G2	2	10
PBoV-G3	4	10
PDCoV	4	6
PEDV	6	15
PoRV	4	10
TGEV	4	10
SADS-CoV	4	8
*Salmonella*	2	9
IC	2	10

^*a*^ PDCoV, porcine deltacoronavirus; PEDV, porcine epidemic diarrhea virus; TGEV, transmissible gastroenteritis virus; SADS-CoV, swine acute diarrhea syndrome coronavirus; PoRV, porcine rotavirus; HEV, hepatitis E virus; PBoV, porcine bocavirus; PBoV-G1/G2/G3, porcine bocavirus group 1/2/3; IC, internal control.

### Analytical specificity

**Fig 3:**
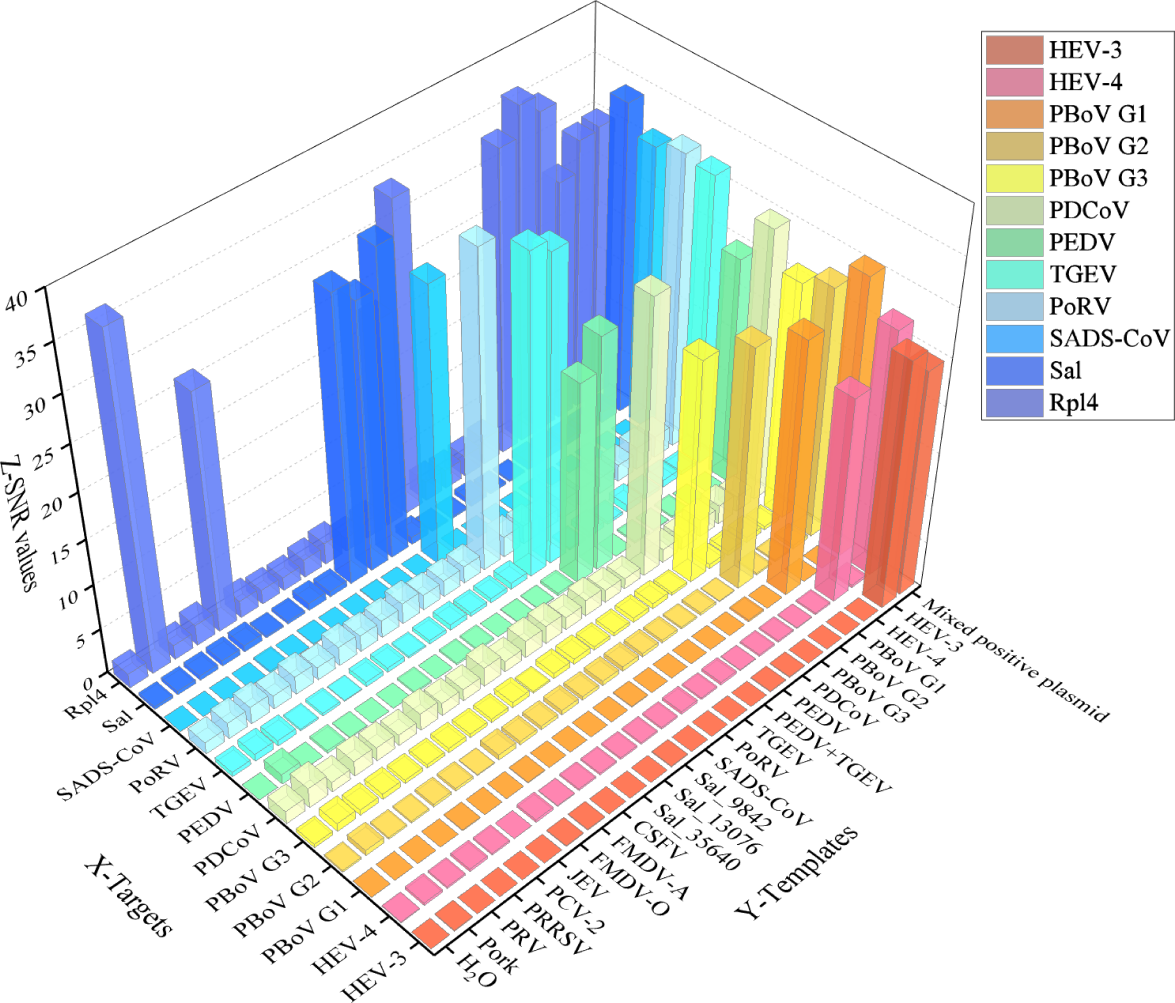
Validation of the analytical specificity of MALDI-TOF NAMS by detecting target and non-target pathogens. With negative and positive controls established, all corresponding targets were detected (SNR ≥ 22.1), and all non-targets were not detected (SNR ≤ 2.2). X-axis: specificity targets for primers and UEP used in this assay. Y-axis: mixed standard plasmid (positive control), target and non-target pathogen samples, pork (negative control), and water (blank control). Z-axis: signal-to-noise ratio (SNR) values UEP, un-extended probe;SNR, Signal-to-noise ratio; PDCoV, porcine deltacoronavirus; PDCoV, porcine deltacoronavirus; PEDV, porcine epidemic diarrhea virus; TGEV, transmissible gastroenteritis virus; SADS-CoV, swine acute diarrhea syndrome coronavirus; PoRV, porcine rotavirus; HEV-3/4, hepatitis E virus genotype 3/4; PBoV, porcine bocavirus; PBoV-G1/G2/G3, porcine bocavirus group 1/2/3; *Sal, Salmonella*; CSFV, classical swine fever virus; FMDV, foot-and-mouth disease virus; JEV, Japanese encephalitis virus; PCV-2, porcine circovirus 2; PRRSV, porcine reproductive and respiratory syndrome virus; PRV, pseudorabies virus.

Using genomic DNA or cDNA from 10 target pathogens and five non-target controls as templates, the extension reaction products were analyzed via mass spectrometry after amplification. As shown in Fig. 3, SEP peaks corresponding to PDCoV, PEDV, TGEV, SADS-CoV, HEV, PoRV, PBoV-G1/G2/G3, and Sal are exclusively detected in reactions using their respective target pathogens or mixed plasmids as templates. In addition, depending on the extended bases, HEV-3 and HEV-4 are able to be distinguished by a single UEP (Fig. 1A). In contrast, no amplification occurred for non-target pathogens (including CSFV, FMDV, PRRSV, PRV, and PCV 2) or blank controls. The mass spectra of these non-target samples displayed only UEP peaks, confirming the high specificity of the MALDI-TOF NAMS assay for the selected target pathogens. This specificity ensures that the assay can reliably differentiate target pathogens within complex biological samples without cross-reactivity from non-target microorganisms, highlighting its robustness and accuracy in diagnostic applications.

### Analytical sensitivity

The analytical sensitivity of the MALDI-TOF NAMS assay was evaluated using serially diluted mixed plasmids at concentrations ranging from 100 copies/μL to 1.6 copies/μL. All targets were reliably detected at 12.5 copies/μL. Probit regression analysis was then performed to calculate the LoD (99% detection probability) and its 95% confidence intervals based on 10 replicates at each concentration. The results, shown in Table 4, indicate that the LoD values for all targets ranged from 12.20 copies/μL to 33.59 copies/μL, highlighting the assay’s high sensitivity and reliable performance at low nucleic acid concentrations.

**TABLE 4:** Results of sensitivity test^*a*^

Target	Concentration (copies/µL)	
	LoD^*b*^ (99% probability)	95% confidence level
HEV	22.36	14.93 ~ 77.15
PBoV-G1	14.37	10.71 ~ 45.40
PBoV-G2	17.16	13.16 ~ 45.88
PBoV-G3	**33.59**	22.87 ~ 107.01
PDCoV	17.77	12.43 ~ 60.05
PEDV	28.77	20.83 ~ 134.40
PoRV	14.37	10.71 ~ 45.40
TGEV	14.37	10.71 ~ 45.40
SADS-CoV	16.54	12.45 ~ 41.24
*Salmonella*	13.21	9.68 ~ 87.63
IC	**12.20**	8.55 ~ 52.02

^*a*^ PDCoV, porcine deltacoronavirus; PEDV, porcine epidemic diarrhea virus; TGEV, transmissible gastroenteritis virus; SADS-CoV, swine acute diarrhea syndrome coronavirus; PoRV, porcine rotavirus; HEV, hepatitis E virus; PBoV, porcine bocavirus; PBoV-G1/G2/G3, porcine bocavirus group 1/2/3; IC, internal control.

^*b*^ Limit of detection. Bold values column represent the maximum (33.59) and minimum (12.20) LoD in the sensitivity test.

### Repeatability and reproducibility

The repeatability and reproducibility of the MALDI-TOF NAMS assay were evaluated using mixed plasmid templates at high, medium, and low concentrations. At each concentration, the detection rate for all targets was 100% (20/20 per concentration, totaling 60/60). In two subsequent independent experiments, all targets were also detected with 100% positivity (60/60 per batch).

### Evaluation of the MALDI-TOF NAMS on three types of samples

**Fig 4:**
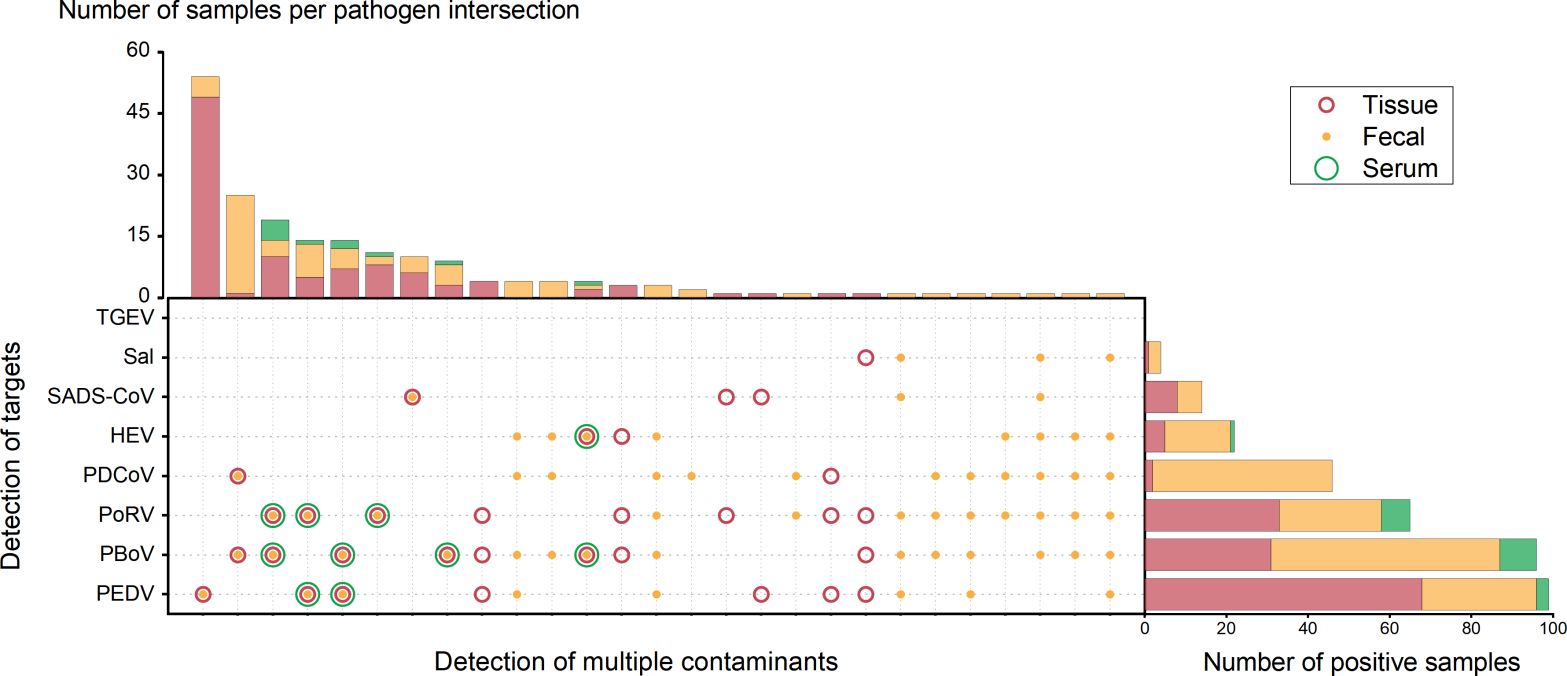
Results from the detection of nucleic acids in 242 samples using MALDI-TOF NAMS. The summary plot illustrates the various pathogen detection types, with different icons representing the three sample types (tissue, fecal, and serum). The upper bar chart shows the number of samples detected for each pathogen type, while the left bar chart represents the number of detections for each pathogen. PBoV-G1/G2/G3 was combined as a single category for PBoV, and HEV-3/HEV-4 was grouped as HEV for statistical purposes. PDCoV, porcine deltacoronavirus; PEDV, porcine epidemic diarrhea virus; TGEV, transmissible gastroenteritis virus; SADS-CoV, swine acute diarrhea syndrome coronavirus; PoRV, porcine rotavirus; HEV, hepatitis E virus; PBoV, porcine bocavirus; *Sal, Salmonella*.

**Fig 5:**
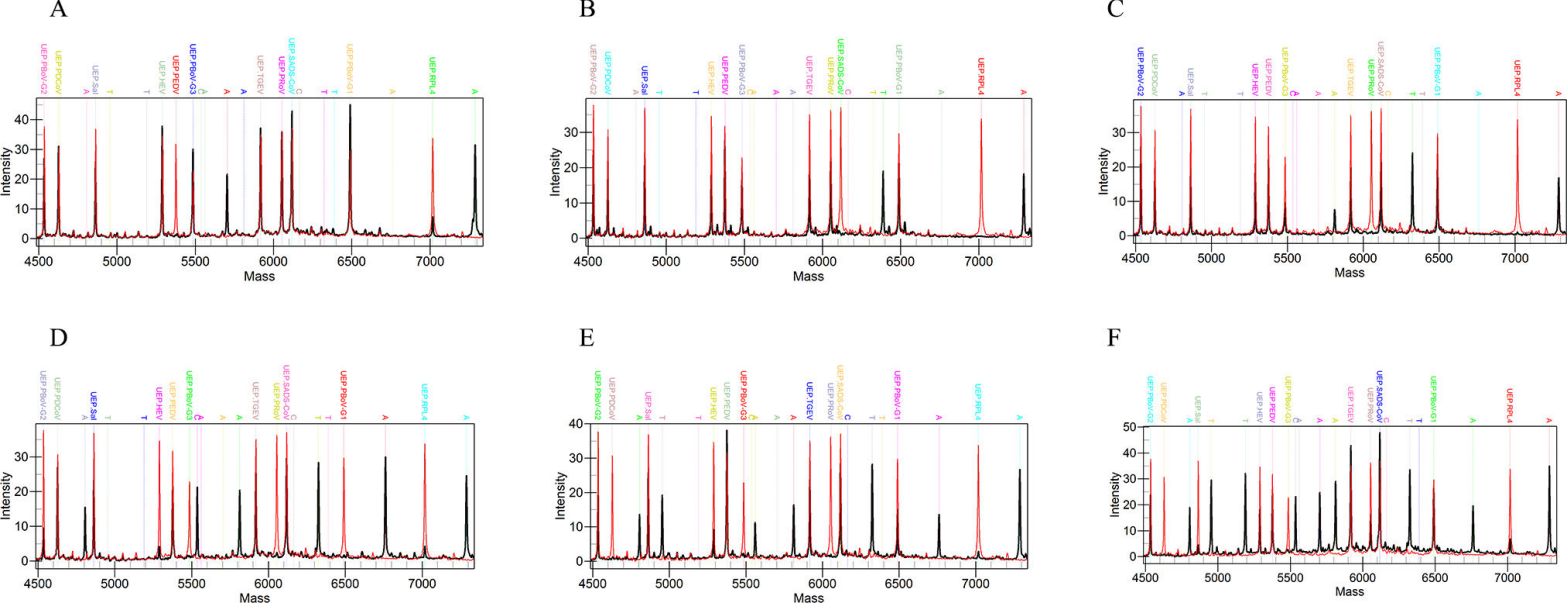
MALDI-TOF NAMS results of selected clinical samples. (**A**) and (**B**) show tissue samples with positive detection of PEDV and SADS-CoV, respectively. (**C**) presents a blood sample detecting both PoRV and PBoV-G3. (**D**), (**E**), and (**F**) represent fecal samples: (**D**) shows detection of HEV-4, PoRV, and PBoV-G1/G2/G3; E detects HEV-3, PDCoV, PoRV, and PBoV-G1/G2/G3; and (**F**) identifies HEV-4, PDCoV, PEDV, PoRV, PBoV-G1/G2/G3, and *Salmonella*. PDCoV, porcine deltacoronavirus; PEDV, porcine epidemic diarrhea virus; SADS-CoV, swine acute diarrhea syndrome coronavirus; PoRV, porcine rotavirus; HEV-3/4, hepatitis E virus genotype 3/4; PBoV-G1/G2/G3, porcine bocavirus group 1/2/3.

The performance of the MALDI-TOF NAMS method was further validated using 242 clinical samples, including feces (*n* = 97), tissue (*n* = 132), and serum (*n* = 13) specimens. Overall, 192 samples tested positive for at least one target pathogen. Notably, while TGEV was not detected in any sample type, all other targets were identified to varying degrees (Fig. 4; Table 5). The results of clinical samples showed the distinct SEP peaks corresponding to detected pathogens (Fig. 5).

**TABLE 5:** Results of 242 clinical samples tested using NAMS and their qPCR compliance^*a*^

Target	Fecal (97)		Tissue (132)		Serum (13)		Total (242)		Clinical sensitivity^*b*^	Clinical specificity^*c*^	Total agreement^*d*^
	NAMS positive	qPCR positive	NAMS positive	qPCR positive	NAMS positive	qPCR positive	NAMS positive	qPCR positive			
PDCoV	44 (45.4%)	42 (43.3%)	2 (1.5%)	2 (1.5%)	0 (0.0%)	1 (7.7%)	46	45	88.9%	97.0%	95.5%
PEDV	28 (28.9%)	24 (24.7%)	68 (51.5%)	68 (51.5%)	3 (23.1%)	3 (23.1%)	99	95	92.6%	92.5%	92.6%
TGEV	0 (0.0%)	1 (1.03%)	0 (0.0%)	0 (0.0%)	0 (0.0%)	0 (0.0%)	0	1	0.0%	100.0%	99.6%
SADS-CoV	6 (6.2%)	4 (4.1%)	8 (6.1%)	10 (7.6%)	0 (0.0%)	0 (0.0%)	14	14	85.7%	99.1%	98.3%
PoRV	25 (25.8%)	22 (22.7%)	33 (25.0%)	32 (24.2%)	7 (53.8%)	6 (46.2%)	65	60	96.7%	96.2%	96.3%
HEV	16 (16.5%)	5 (5.2%)	5 (3.8%)	4 (3.0%)	1 (7.7%)	1 (7.7%)	22	10	90.0%	94.4%	94.2%
HEV-3	6 (6.2%)	/	0 (0.0%)	/	0 (0.0%)	/	6	/	/	/	/
HEV-4	10 (10.3%)	/	5 (3.8%)	/	1 (7.7%)	/	16	/	/	/	/
PBoV	56 (57.7%)	58 (59.8%)	31 (23.5%)	30 (22.7%)	9 (69.2%)	4 (30.8%)	96	92	95.7%	94.7%	95.0%
PBoV-G1	54 (55.7%)	49 (50.5%)	31 (23.5%)	28 (21.2%)	7 (53.8%)	7 (53.8%)	92	84	98.8%	94.9%	95.9%
PBoV-G2	22 (22.7%)	21 (21.6%)	11 (8.3%)	10 (7.6%)	5 (38.5%)	4 (30.8%)	38	35	88.6%	96.6%	95.5%
PBoV-G3	35 (36.1%)	31 (32.0%)	10 (7.6%)	10 (7.6%)	4 (30.8%)	7 (53.8%)	49	48	85.4%	95.9%	93.8%
*Salmonella*	3 (3.1%)	3 (3.1%)	1 (0.8%)	1 (0.8%)	0 (0.0%)	0 (0.0%)	4	4	100.0%	100.0%	100.0%
Total^*e*^	/	/	/	/	/	/	429	396	93.4%	96.9%	96.0%

^*a*^ PDCoV, porcine deltacoronavirus; PEDV, porcine epidemic diarrhea virus; TGEV, transmissible gastroenteritis virus; SADS-CoV, swine acute diarrhea syndrome coronavirus; PoRV, porcine rotavirus; HEV, hepatitis E virus; HEV-3/4, hepatitis E virus genotype 3/4; PBoV, porcine bocavirus; PBoV-G1/G2/G3, porcine bocavirus group 1/2/3. For HEV3 and HEV4, no Ct values were detected by qPCR in all three types of samples, the results are represented by "/".

^*b*^ Clinical sensitivity = TP / (TP + FN); TP, true positives; FN, false negatives.

^*c*^ Clinical specificity = TN / (TN + FP); TN, true negatives; FP, false positives.

^*d*^ Overall agreement = (TP + TN) / Total; TP, true positives; TN, true negatives; Total, total sample size.

^*e*^ The total compliance rate is calculated for PBoV by the results of G1, G2 and G3 typing tests.

To assess the accuracy of MALDI-TOF NAMS, qPCR was used as a reference method, and the concordance rates between the two methods were analyzed (Table 5). The statistics for HEV include HEV-3 and HEV-4, and PBoV includes the combined results for all genotypes. The results revealed that the total concordance rates for individual targets ranged from 92.6% to 100.0% (except TGEV). When data for all targets were combined, the total concordance rates for different sample types ranged from 93.2% to 98.3%, with an overall concordance rate of 96.2% for all 242 samples. These findings demonstrate a high level of agreement between MALDI-TOF NAMS and qPCR, supporting the reliability and accuracy of the newly developed assay for detecting multiple pathogens in clinical diarrhea samples.

## DISCUSSION

Porcine diarrhea syndrome (PDS) disease poses a serious threat to the health of pig herds and the development of the pig industry worldwide, leading to significant economic losses, especially in the import and export trade (21). The complex landscape of diarrheal pathogens in pigs, coupled with the emergence of novel pathogens, has heightened the challenge of disease prevention and control. Thus, there is an urgent need for new diagnostic techniques capable of addressing mixed infections and detecting a wide range of pathogens efficiently. This study aimed to evaluate the performance of the nucleic acid mass spectrometry (NAMS) method in detecting pathogens associated with PDS. Our findings demonstrated the capability of NAMS to achieve high-throughput, multiplex pathogen detection with high sensitivity and specificity, addressing some of the limitations of existing diagnostic methods, such as ELISA, qPCR, and NGS. These results underscore the potential of NAMS as a robust tool for large-scale pathogen surveillance and disease control.

Our analysis of 242 samples revealed an overall positivity rate of 81.4%, 77.3%, and 84.6% in fecal, tissue, and serum samples, respectively (Table 5). Notably, mixed infections were prevalent, with 39.2% of positive samples exhibiting co-detection of multiple pathogens. The proportion of multiple detections reached 61.9% (60/97) in fecal samples, 27.3% (36/132) in tissue, and 69.2% (9/13) in serum. Specifically, our findings revealed that HEV and *Salmonella* were exclusively detected in mixed infections, suggesting their potential role in exacerbating disease severity. Moreover, PBoV, frequently detected in co-infections with PDCoV (41 cases), PoRV (36 cases), and PEDV (29 cases), emerged as a key player in mixed infections, consistent with its known role as an auxiliary pathogen (6). These findings emphasize the complexity and diversity of pathogen interactions in the context of porcine diarrheal syndromes.

From the results of individual viruses, PEDV, the main pathogen of porcine diarrhea outbreaks, had a significantly higher positive rate than other pathogens. In this study, the positivity rate for PEDV was as high as 40.9% (99/242), with single-pathogen infections predominantly observed in tissue samples (90.7%, 49/54). This aligns with reports of PEDV’s high virulence and association with severe outbreaks in piglets (22). In addition, studies have shown that the use of commercial vaccines may accelerate the evolution of PEDV, resulting in multiple PEDV infections in the same pig farm (23). This highlights the severity of PEDV epidemics and the urgency for improved immunization and prevention strategies. As the other major causative virus of porcine diarrhea, PDCoV was primarily detected in fecal samples (95.7%, 44/46). Clinical symptoms of PDCoV infection resemble those of PEDV and TGEV but are less severe in piglets (24). Therefore, PDCoV often co-infects with other pathogens, such as PEDV and PoRV (25). This was confirmed in the present study, where the co-infection rates of PDCoV with PEDV and PoRV were 21.7% and 23.9%, respectively. PoRV infections, unlike PEDV and TGEV, generally cause sporadic diarrhea with lower mortality (26). Recent investigations (2022–2023) reported that the positivity rate of PoRV in Chinese pig farms reached 86.5%, with a sample positivity rate of 51.2% (27). In this study, PoRV was detected across all three sample types, with positivity rates of 25.8% (25/97) in fecal samples and 25.0% (33/132) in tissues, both lower than previously reported data. However, PoRV positivity rose to 53.8% (7/13) in sera, potentially due to the small sample size of serum tested.

The SADS-CoV was detected at a low frequency in this instance (5.8%, 140/242), mainly as a single pathogen (71.4%, 10/14). This may be related to its sporadic regional distribution, and as a diarrheal pathogen with a high mortality rate, further surveillance is needed (28, 29). TGEV was once a major pathogen causing diarrhea in pigs, leading to significant economic losses in the global swine industry. The absence of TGEV in this study corroborates reports of its reduced prevalence due to the widespread circulation of its natural deletion mutant strain, PRCV (30, 31). To minimize interference from PRCV in detection, the TGEV target region in this study was designed within the PEDV S gene region where PRCV exhibits a deletion. This strategy will effectively enhance detection specificity and reduce the impact of PRCV on the diagnosis of diarrhea-associated pathogens. It should be noted that in qPCR validation, one fecal sample tested positive for TGEV/SADS-CoV (Ct = 36.2/18.7), with only SADS-CoV detected by the NAMS method. A later digital droplet PCR (ddPCR) confirmed TGEV/SADS-CoV positivity (positive/total droplet count = 29/262,400). Earlier specificity testing with TGEV inactivated strains (WH-1R) and bivalent PEDV/TGEV live vaccines (WH-1R + AJ1102R) consistently yielded expected positive results. In addition, all the remaining samples were verified as TGEV-free using qPCR. Thus, the absence of TGEV detection in this sample is likely due to its proximity to the NAMS detection limit (10.71–45.40, 95% confidence level). This limitation does not compromise the reliability of TGEV detection in most cases of porcine diarrhea. All *Salmonella*-positive samples were associated with co-detection of multiple pathogens, a scenario frequently encountered in fecal samples from complex composite infections (8).

Beyond the major diarrheal pathogens, PBoV exhibited a high overall detection rate (39.7%) across all sample types. Among the positive cases, the G1 genotype of PBoV was the most frequently detected (23.5%–55.7%), consistent with previous findings (4). While the pathogenic mechanism of PBoV remains unclear, studies indicate frequent co-infections with other pathogens, such as PRRSV, PCV2, and CSFV, with rising prevalence in recent years (6, 32). PBoV has also been detected in both clinically healthy pigs and those with respiratory or enteric diseases worldwide, leading some researchers to propose that PBoV may act as an auxiliary virus rather than a primary pathogen (6). In this study, co-infection rates of PBoV with other diarrhea-related pathogens were 38.0%, significantly higher than the 1.2% in healthy pigs, suggesting a potential role for PBoV in porcine diarrhea. Additionally, a substantial proportion of samples contained two or more PBoV genotypes, indicating widespread cross-infections among PBoV subtypes in hosts. Previous studies have also demonstrated that multiple PBoV genotypes frequently co-circulate, with individuals often simultaneously infected with several PBoV strains (32, 33). The potential of PBoV for cross-species transmission to humans further underscores its significance as a potential public health concern (7). The detection rate of HEV (9.1%) was much lower than that of PBoV for the same severe mixed infections. HEV-3 was exclusively detected in fecal samples, while HEV-4 was identified across all sample types, with detection rates ranging from 3.8% to 10.3%. As a zoonotic pathogen with a broad host range, the correlation between HEV prevalence and multiviral co-infections emphasizes the need for targeted surveillance on diarrhea-endemic farms (9, 34).

PEDV, TGEV, SADS-CoV, and PDCoV all belong to the *Coronaviridae* family. Coronaviruses have garnered significant attention due to their broad host range and potential for cross-species transmission (2, 35). The emergence of coronaviruses, such as MERS-CoV, SARS-CoV, and SARS-CoV-2, serves as a stark reminder of the risks associated with inter-species spillover (36, 37). For instance, PDCoV, first identified in 2012, is thought to have originated from an interspecies transmission event involving avian and mammal coronaviruses, as suggested by phylogenetic analyses. Research has shown that PDCoV utilizes its spike (S) protein to bind with host aminopeptidase N (APN), enabling it to infect cells from pigs, chickens, and humans. The highly conserved nature of APN across species likely plays a critical role in facilitating PDCoV’s cross-species infectivity (35). Notably, PDCoV is not the only enteric coronavirus that leverages APN. PEDV and TGEV also depend on this receptor, suggesting a potential commonality in the mechanisms underlying host switching in coronaviruses (37, 38). Moreover, studies indicate that SADS-CoV can effectively replicate in various mammalian cell lines, including primary human lung and intestinal cells, further underscoring the potential for interspecies transmission (28). Similar to HEV and PBoV, the widespread distribution and zoonotic potential of swine-origin coronaviruses necessitate comprehensive investigations into their epidemiology and transmission dynamics. Such efforts are critical for assessing and mitigating potential public health risks associated with these pathogens.

**Fig 6:**
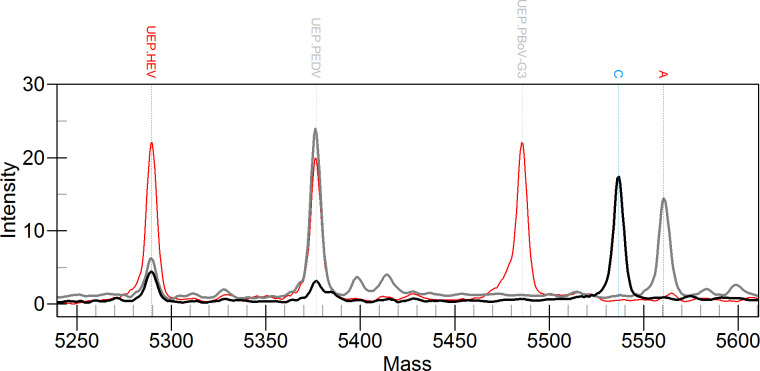
MALDI TOF NAMS was able to distinguish between HEV-3 and HEV-4 types. Results from different HEV-positive samples showed no interference between SEPs for HEV-3 (A) and HEV-4 (C). HEV-3/4, hepatitis E virus genotype 3/4.

In this study, the advantages of NAMS were utilized for SNP genotyping of HEV-3 and HEV-4 genes (Fig. 6). Similarly, SNP genotyping can be further employed to distinguish between pathogen variants, such as virulent versus attenuated strains and wild-type versus vaccine strains. On the other hand, the successful detection of the *Salmonella* target demonstrates that the NAMS method can simultaneously detect RNA viruses, DNA viruses, and bacteria. This significantly broadens the scope of pathogen detection and its application scenarios, particularly for diagnosing syndromes with unknown etiologies. This capability relies on selecting an appropriate nucleic acid extraction method. In future research, leveraging the scalability of targets in the NAMS method, we can further expand its application to include bacterial pathogens causing porcine diarrhea, aiming to establish a more comprehensive diagnostic system for porcine digestive tract diseases. It should be noted that, due to the relatively small number of clinical samples collected, the distribution of certain pathogens may only reflect regional epidemiological characteristics and farm management practices and may not fully reflect the diversity of cases in the real world (e.g., *Salmonella* and hepatitis E virus).

Although portable mass spectrometry devices have been proposed, current MALDI-TOF MS platforms remain relatively large and laboratory-based, making this method better suited to centralized surveillance rather than true field deployment. From a cost–benefit perspective, while the instrumentation is relatively costly and may not outperform conventional qPCR in small-scale testing, the MALDI-TOF NAMS approach provides clear advantages in high-throughput, multiplex applications. The platform can analyze up to ~40 targets simultaneously (39), and the use of unmodified UEPs enables low-cost panel expansion, improving the economics per target as panel size and sample volume increase. These features make the method particularly advantageous for routine monitoring, mixed-infection detection, and comprehensive pathogen surveillance in centralized laboratory settings. Future advances in miniaturized MS technology may further enhance its applicability in resource-limited or field environments.

### Conclusion

In conclusion, this study demonstrates the potential of NAMS as a promising tool for the detection and genotyping of swine diarrhea pathogens. While the method shows strong sensitivity and specificity, it remains an emerging technology that complements traditional diagnostic approaches, such as qPCR and NGS. The ability to perform multiplexed detection of these pathogens in a single assay not only enhances diagnostic accuracy but also allows for a comprehensive understanding of co-infections, which are common in clinical settings. This is particularly useful for complex mixed infections, where traditional single-target approaches tend to complicate diagnosis. The broader potential of NAMS lies in its application to pathogen monitoring, especially in epidemiological surveillance, early detection of emerging infectious diseases across animal populations, and its possible integration into entry-exit quarantine processes. It provides a new means for more efficient and accurate pathogen screening, which is vital for controlling the spread of animal diseases across borders.

## Data Availability

The raw data supporting the findings of this study have been deposited in Zenodo under DOI: https://doi.org/10.5281/zenodo.17181630. All data are publicly available.

## SUPPLEMENTAL MATERIAL

Supplemental material spectrum.01328-25-s0001.docx Table S1; Figures S1 and S2.

ASM does not own the copyrights to Supplemental Material that may be linked to, or accessed through, an article. The authors have granted ASM a non-exclusive, world-wide license to publish the Supplemental Material files. Please contact the corresponding author directly for reuse.
