# Supplementary material for: Multiplex detection and application of MALDI-TOF NAMS for porcine diarrheal pathogens
Source: Microbiol Spectr. 2025 Oct 27;13(12):e01328-25. doi: 10.1128/spectrum.01328-25 (PMC12671185; doi:10.1128/spectrum.01328-25)
Supplement: Supplemental material — Table S1; Figures S1 and S2. [file spectrum.01328-25-s0001.docx]

**Supplemental material**

**Table S1.** *Salmonella* and PBoV primers used for qPCR*^a^*

| Primer | Primer sequence (5′→3′) | Expected product (bp) |
| --- | --- | --- |
| Sal_F | GCTGCTTTCTCTACTTAAC | 95 |
| Sal_R | GTAATGGAATGACGAACAT |  |
| PBoV-G1_F | TGAGCTAATCCCTGAACTG | 94 |
| PBoV-G1_R | GTCTGAGCCTGTATCACCTAT |  |
| PBoV-G2_F | GGGCACTGATTATATCTTTAC | 86 |
| PBoV-G2_R | CCCTGACATCTTTCCATT |  |
| PBoV-G3_F | ACTCTTTGCAGTCTCTGACTCTTC | 108 |
| PBoV-G3_R | GTTCCCCCGTGTCTTTAG |  |

*^a^*Sal, *Salmonella*; PBoV-G1/G2/G3, porcine bocavirus group 1/2/3.


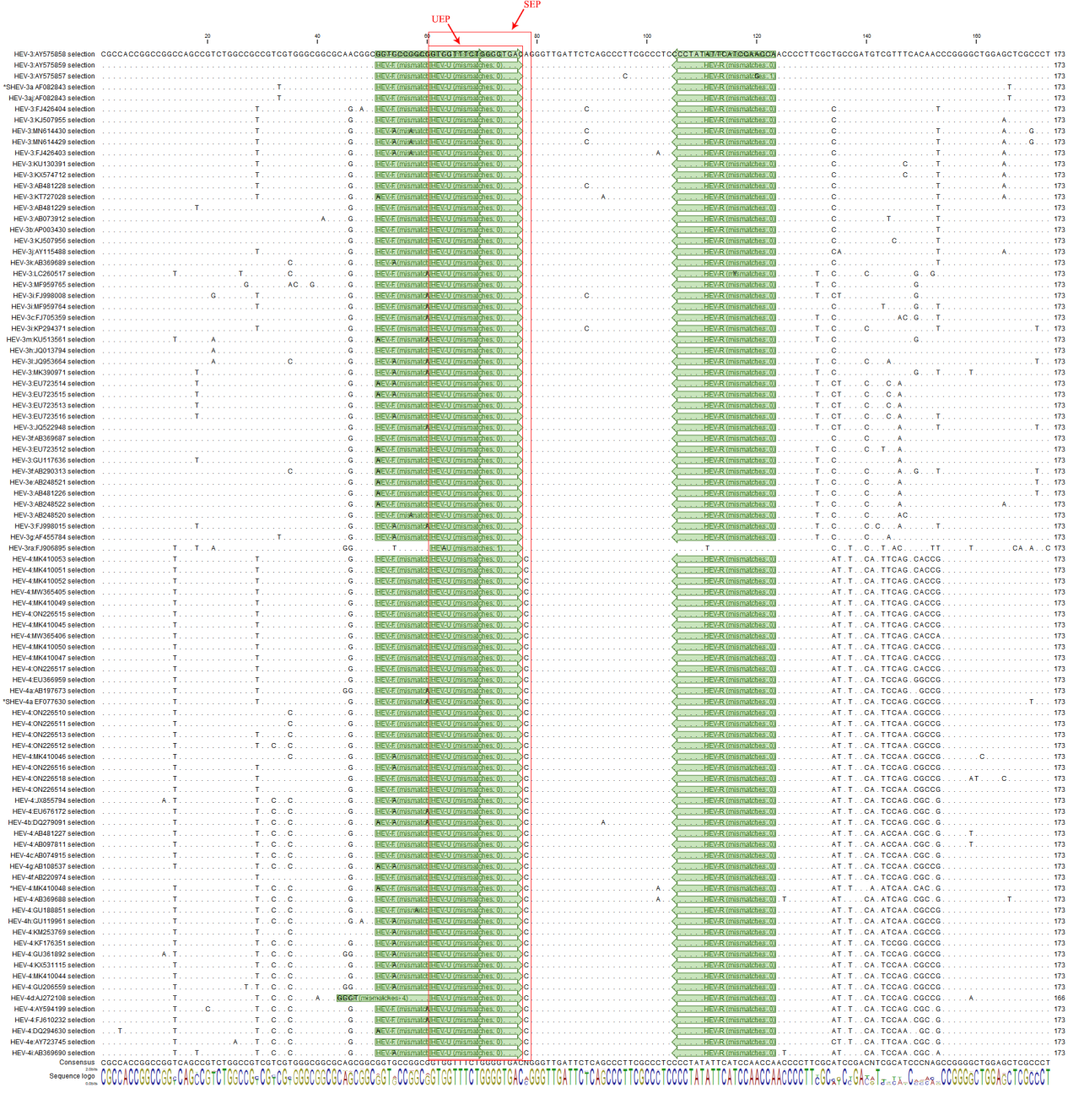


**Fig. S1.** Schematic diagram of primer and UEP positions in the HEV-3 and HEV-4 sequences. UEP, un-extended probe; SEP, single-base extended products; HEV-3/4, hepatitis E virus genotype 3/4.


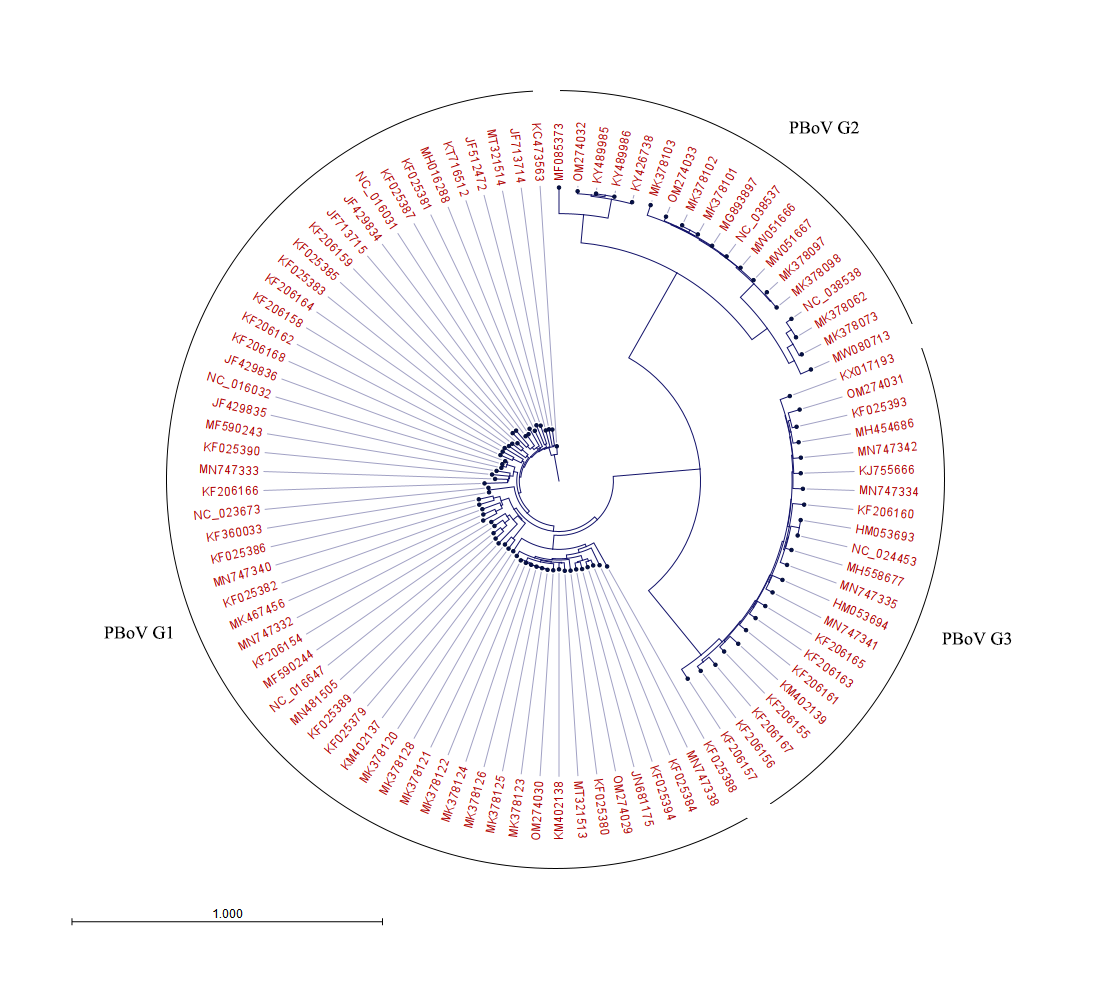


**Fig. S2.** Whole-genome phylogenetic analysis of PBoVs (neighbor-joining, 1,000 bootstraps). PBoV-G1/G2/G3, porcine bocavirus group 1/2/3.
